# COVID-19 and Pre-existing Type 2 Diabetes Mellitus: A Retrospective Observational Study From Eastern India on the Association Between Glycaemic Control and Treatment Outcomes

**DOI:** 10.7759/cureus.35165

**Published:** 2023-02-19

**Authors:** Poulomi Mukherjee, Soumyabrata RoyChaudhuri, Anirban Majumder

**Affiliations:** 1 Community Medicine, Kolkata Medical College & Hospital, Kolkata, IND; 2 Endocrinology, Kali Prasad Chowdhury Medical College & Hospital, Kolkata, IND

**Keywords:** treatment outcome, type 2 diabetes mellitus, hospital mortality, hba1c, glycaemic control, covid-19

## Abstract

Background: Diabetes has emerged as an important risk factor for causing severe illness and death from COVID-19. There is a paucity of structured data from the Indian subcontinent on the impact that glycaemic control (both immediate and remote) has on the degree of required medical intervention and mortality among hospitalized COVID-19 patients with type 2 diabetes mellitus (T2DM).

Objectives: To evaluate the differences in clinical characteristics and treatment outcomes between well-controlled and poorly controlled patients with T2DM and COVID-19.

Methods: This was a retrospective observational study. Data on 177 patients who were hospitalized between February 2021 and July 2021 were categorized into four groups using a cut-off admission plasma glucose of <200 mg/dL and glycated hemoglobin (HbA1c) <7.5%.

Results: Patients with poorly controlled diabetes presented at a significantly older age than the other groups. Radiological findings suggested severe lung involvement in them. As a combined group patients with HbA1c ≥7.5% required more ventilatory requirement as compared with the group having HbA1c <7.5% irrespective of admission glucose. They also required prolonged hospitalization and intensive care unit (ICU) stays as compared with the well-controlled diabetes group. In this study, within similar ranges of HbA1c admission glucose seemed to have a numerical impact on mortality without being able to achieve statistical significance.

Conclusion: From the current study, it can be concluded that poor glycaemic control, particularly HbA1c ≥7.5%, is an important risk factor for the development of severe COVID-19 and a predictor for the requirement of more intensive treatment and adverse treatment outcomes leading to increased hospital and ICU stay.

## Introduction

Severe acute respiratory syndrome coronavirus 2 (SARS-CoV-2), the novel coronavirus that causes coronavirus disease 2019 (COVID-19), was first reported in Wuhan, China, in December 2019 and has spread worldwide. As of 11 April 2022, 497,057,239 globally confirmed cases of COVID-19 have been reported on the World Health Organization COVID-19 dashboard, including 6,179,104 deaths [1].

Studies have suggested that most people affected by COVID-19 have comorbidities, the most prevalent of which are hypertension, diabetes mellitus, and cardiovascular disease [2-5]. Generally, about 10-20% of patients with COVID-19 had diabetes. Research suggests that diabetic patients were more susceptive to SARS-CoV-2 and in the long run, had poor disease-related outcomes [6-8]. In diabetic patients glucotoxicity coupled with endothelial damage by inflammation, oxidative stress, and cytokine production contribute to an increased risk of thromboembolic complications and damage to vital organs [9]. Moreover, drugs commonly used in the treatment of patients with COVID-19, such as systemic corticosteroids or antiviral agents, might aggravate hyperglycemia.

The cornerstone for diabetes management is glucose control. Some researchers have clarified the importance and provided insights for glucose control in patients with diabetes and COVID-19 [10-12]. For individuals with COVID-19 and pre-existing diabetes, a key challenge for clinicians is to improve outcomes in the face of uncertainty regarding the degree of glycaemic management that should be maintained and any effects this might have on the benefits and risks of the overall treatment. Thus, detailed analysis of data from such patients is needed that links glycaemic control with treatment outcomes, including mortality. The prevalence of type 2 diabetes mellitus (T2DM) in India is high, and the paucity of data on its association with COVID-19 warrants the identification of factors responsible for severe outcomes in such patients. The present study included COVID-19 patients with pre-existing T2DM admitted to a tertiary care hospital in Kolkata, West Bengal focusing on the association between their glycaemic control and treatment outcomes.

## Materials and methods

Study design

This retrospective observational study evaluated T2DM patients hospitalized at Kali Prasad Chowdhury Medical College and Hospital in Kolkata, West Bengal during the second wave of the COVID-19 pandemic between February 2021 and July 2021 with laboratory-confirmed COVID-19 illness. The objective of the study was to find out whether good glycaemic control at the time of admission is associated with better treatment outcomes for COVID-19. We used a cut-off admission plasma glucose of <200 mg/dL and HbA1c <7.5% as a cut-off for grouping the patients' data. So there were four groups based on their respective glycaemic control:

Group (I) - T2DM patients with HbA1c <7.5% and admission glucose <200 mg/dL

Group (II) - T2DM patients with HbA1c <7.5% and admission glucose ≥200 mg/dL

Group (III) - T2DM patients with HbA1c ≥7.5% and admission glucose <200 mg/dL

Group (IV) - T2DM patients with HbA1c ≥7.5% and admission glucose ≥200 mg/dL

The patients were initially admitted to the Covid ward of the hospital and afterward, patients with warning signs were transferred to the intensive care unit (ICU). The management protocol of the patients followed in the study was as per the guideline approved by the department of health and family welfare, the government of West Bengal [13]. All patients were given insulin to control blood sugar.

Ethical considerations

The study being a retrospective observational study ethical approval was not sought. Moreover, due to the retrospective nature of the study, participants were de-identified and could not be contacted hence informed consent was not obtained. Confidentiality and anonymity were maintained. This study is solely intended for academic purposes. All tenets of Helsinki's declaration on bioethics policy were adhered to.

Definitions

As per WHO interim guidelines reverse transcriptase polymerase chain reaction (RT-PCR) positive patients (sample source nasal and pharyngeal swab) were considered to be confirmed COVID-19-positive cases. Patients who required a hospital stay greater than 24 hours were taken as hospitalized patients [14]. The first glucose measurement within a time window of 4 hours before and up to 4 hours after admission was used as admission glucose and categorized as well-controlled (< 200 mg/dL) and poorly controlled (≥200mg/dL) [15,16]. Moreover, according to the National Institute for Health and Care Excellence (NICE) guidelines for the management of type 2 diabetes in adults, we selected HbA1c value <7.5% (58 mmol/mol) as a criterion for good glycaemic control [17].

Outcome measures

Data for four groups of patients were compared against each other to assess differences in (1) demographic and clinical characteristics like age, gender, and radiological findings using an average visual score from CT severity score (CTSS) from high-resolution computed tomography (HRCT) chest (assigned out of 25 based upon percentage area involved in each of the five lobes); (2) treatment outcome including ICU stay, length of hospital stay, the requirement of ventilator support and death.

Statistical analysis

In the present study, we carried out descriptive and inferential statistical analysis. Results on continuous variables were presented as mean (SD) for parametric data. The normality of data was tested by the Anderson-Darling test, Shapiro-Wilk, Kolmogorov-Smirnoff test, and visually by QQ plot. The variance was tested by Levene’s test. For data measured in ratio scales like CTSS, ICU stay, and hospital stays, the analysis of covariance (ANCOVA) test was used to find the difference between the groups, and post hoc Dunnett’s test or Bonferroni test was carried out to find the inter-group differences. For categorical variables, the difference between groups was tested by the Chi-square test. Fischer's exact approach was used for post hoc analysis of a Chi-squared test. Statistical evaluations carried out were two-sided and the cut-off for statistical significance was taken as p <0.05.

Statistical software

The statistical software namely SAS (Statistical Analysis System) version 9.2 for windows, SAS Institute Inc. Cary, NC, USA and Statistical Package for Social Sciences (SPSS Complex Samples) version 21.0 for windows, SPSS, Inc., Chicago, IL, USA, has been used for data analysis.

## Results

In total, there were 177 patients with pre-existing T2DM and COVID-19 included in this study. Thirty-two patients (18.1%) had HbA1c <7.5% and admission glucose <200 mg/dL (Group-I), 36 patients (20.34%) had HbA1c <7.5%, and admission glucose ≥200 mg/dL (Group-II). Forty-seven patients (26.6%) had HbA1c ≥7.5% and admission glucose <200 mg/dL (Group-III) and 62 patients (35.03%) had HbA1c ≥7.5% and admission glucose ≥200 mg/dL(Group-IV).

The age distribution of the participants is shown in Table 1. Overall the mean age of the patients was 53.75 years (standard deviation of 14.02 years). There was a significant difference in mean age across four groups of patients (ANOVA, p = 0.004). Inter-group analysis was done by the Bonferroni test. Patients with HbA1c ≥7.5% + admission glucose ≥200 mg/dL - (Group-IV i.e poor glycaemic control) tended to be significantly older compared to patients of Group-I having HbA1c <7.5 either with admission glucose <200 mg/dL (p = 0.008) or of Group-II with admission glucose ≥200 mg/dL (p = 0.031). Besides significant age difference was found between patients having HbA1c ≥7.5% and patients having HbA1c <7.5% both with admission glucose <200 mg/dL (p = 0.031) viz Group-III versus Group-I. A total of 110 patients (62.15%) were male and 67 patients (37.85%) were female, however, with respect to gender there was no statistically significant difference between the groups (Chi-square, p = 0.0529).

**Table 1:** Distribution of the study participants by age, N=177 * p computed by ANOVA test; p<0.05 considered as statistically significant. Post hoc test performed by Bonferroni test. a - difference between Groups I and II, b - difference between Groups I and III, c - difference between Groups I and IV, d - difference between Groups II and III, e - difference between Groups II and IV, f - difference between Groups IV and III T2DM: type 2 diabetes mellitus; ANOVA: analysis of variance

Group	N	Mean	Std. deviation	P (ANOVA)	Post hoc test
Known T2DM + HbA1c<7.5% + Admission glucose<200 mg/dL (I)	32	45.66	15.924	0.004*	a-0.203
					b-0.038*
					c-0.008*
Known T2DM + HbA1c<7.5% + Admission glucose≥200 mg/dL (II)	36	48.54	13.336		d-0.118
Known T2DM + HbA1c≥7.5% + Admission glucose<200 mg/dL (III)	47	53.83	11.781		
Known T2DM + HbA1c≥7.5% + Admission glucose≥200 mg/dL (IV)	62	60.81	11.437		e-0.031*
					f-0.082
Total	177	53.75	14.021		

Table 2 presents the effects of glycaemic control on the severity of COVID-19 infection of patients with T2DM and COVID-19 after controlling for the effects of baseline age and body weight. Three parameters were assessed viz chest CTSS, ICU stay, and length of hospital stay. The mean CTSS was 12.47 (standard deviation 2.76). The mean duration of ICU stay and hospital stay of the patients was 5.36 days (standard deviation of 3.34 days) and 9.94 days (standard deviation of 3.26 days), respectively. There was a statistically significant difference in mean CTSS, mean duration of ICU stay, and mean duration of hospital stay between the four groups of patients (ANCOVA, p = 0.011, <0.001, 0.002, respectively). Post hoc Dunnett’s test showed that compared to the well-controlled group - Group I (HbA1c <7.5% and admission glucose <200 mg/dL), CTSS was significantly higher (p = 0.005) in the poor-controlled group - Group IV (HbA1c ≥7.5% + admission glucose ≥200 mg/dL). A total of 133 patients (75.14%) needed admission to ICU. The length of ICU stay and hospital stay was significantly less in the well-controlled group compared to the groups with poor long-term glycaemic control i.e. HbA1c ≥7.5%. However, within the poorly controlled HbA1c groups, viz Group III and Group IV (HbA1c >7.5%) admission glucose did influence the ICU stay, with higher admission glucose leading to a significantly higher ICU stay. Hospital stay on the other hand was influenced by HbA1c with higher HbA1c requiring significantly more days in hospital management.

**Table 2:** Associations between glycaemic control and clinical characteristics of patients with T2DM and COVID-19 † p computed by ANCOVA test after adjusting for baseline age and bodyweight; post hoc test performed by Dunnett’s test. a - difference between Groups I and II, b - difference between Groups I and III, c - difference between Groups I and IV, d - difference between Groups II and III, e - difference between Groups II and IV, f - difference between Groups IV and III T2DM: type 2 diabetes mellitus; CTSS: chest CT severity score; ICU: intensive care unit; ANCOVA: analysis of covariance

Clinical characteristics	Groups	N	Mean	Std. deviation	Std. error	95% Confidence interval for mean		P value (ANCOVA)	Post hoc test
						Lower bound	Upper bound		
CTSS	Known T2DM + HbA1c<7.5% + Admission glucose<200 mg/dL (I)	32	11.41	1.829	.323	10.75	12.07	0.011*	a-0.583
									b-0.230
									c-0.005*
	Known T2DM + HbA1c<7.5% + Admission glucose≥200 mg/dL (II)	36	12.03	1.543	.261	11.50	12.56		d-0.929
	Known T2DM + HbA1c≥7.5% + Admission glucose<200 mg/dL (III)	47	12.47	2.765	.403	11.66	13.28		
	Known T2DM + HbA1c≥7.5% + Admission glucose≥200 mg/dL (IV)	62	13.27	3.422	.435	12.41	14.14		e-0.091
									f-0.683
	Total	177	12.47	2.761	.208	12.06	12.88		
ICU stay	Known T2DM + HbA1c<7.5% + Admission glucose<200 mg/dL (I)	22	3.50	2.559	.546	2.37	4.63	<0.001*	a-1.000
									b-0.038*
									c<0.001*
	Known T2DM + HbA1c<7.5% + Admission glucose≥200 mg/dL (II)	24	3.50	1.560	.319	2.84	4.16		d-0.006*
	Known T2DM + HbA1c≥7.5% + Admission glucose<200 mg/dL (III)	40	5.70	3.502	.554	4.58	6.82		
	Known T2DM + HbA1c≥7.5% + Admission glucose≥200 mg/dL (IV)	47	6.89	3.383	.493	5.90	7.89		e-0.501
									f<0.001*
	Total	133	5.36	3.340	.290	4.79	5.93		
Hospital stay	Known T2DM + HbA1c<7.5% + Admission glucose<200 mg/dL (I)	32	8.59	2.525	.446	7.68	9.50	0.002*	a-0.990
									b-0.008*
									c-0.023*
	Known T2DM + HbA1c<7.5% + Admission glucose≥200 mg/dL (II)	36	8.91	1.669	.282	8.34	9.49		d-0.009*
	Known T2DM + HbA1c≥7.5% + Admission glucose<200 mg/dL (III)	47	10.87	3.555	.518	9.83	11.92		
	Known T2DM + HbA1c≥7.5% + Admission glucose≥200 mg/dL (IV)	62	10.52	3.686	.472	9.58	11.47		e-0.997
									f-0.701
	Total	177	9.94	3.257	.246	9.46	10.43		

Table 3 presents the results of the associations of glycaemic control and prognostic outcome of the patients. A total of 18 patients (10.17%) died out of which 11 patients (17.7%) were in the poorly controlled group. However, there was no significant difference in mortality among the four groups of patients (Chi-square, p=0.090). Within the two groups of HbA1c greater than 7.5% and less than 7.5%, however, those with admission glucose less than 200mg/dL had numerical supremacy in terms of less mortality which failed to reach statistical significance.

**Table 3:** Associations between glycaemic control and in-hospital mortality in patients with T2DM and COVID-19 ‡ p computed by chi-square test, p<0.05 considered as statistically significant T2DM: type 2 diabetes mellitus

Group	Outcome		Total N (%)	P
	Alive N (%)	Death N (%)		
Known T2DM + HbA1c<7.5% + Admission glucose<200 mg/dL (I)	31 (96.9)	1 (3.1)	32 (100)	0.09
Known T2DM + HbA1c<7.5% + Admission glucose≥200 mg/dL (II)	33 (91.7)	3 (8.3)	36 (100)	
Known T2DM + HbA1c≥7.5% + Admission glucose<200 mg/dL (III)	44 (93.6)	3 (6.4)	47 (100)	
Known T2DM + HbA1c≥7.5% + Admission glucose≥200 mg/dL (IV)	51 (82.3)	11 (17.7)	62 (100)	
Total	159 (89.8)	18 (10.2)	177 (100)	

Glycaemic control was found to have a significant effect on the requirement of more intensive in-hospital treatment like mechanical ventilator support in patients with T2DM and COVID-19 (Chi-square, p=<0.001) (Table 4). A total of 54.8% of patients with poor glycaemic control (HbA1c ≥7.5% + admission glucose ≥200 mg/dL) required ventilation. In a within-group analysis it was seen that ventilator support was applied significantly more frequently to the individuals with poor long-term glycaemic control viz Group III and Group IV (i.e HbA1c ≥7.5%) compared to the well-controlled group viz Group 1 and Group II (i.e HbA1c <7.5%) irrespective of the admission glucose values. Within similar tertiles of HbA1c, however, more patients with admission glucose greater than 200mg/dL required ventilatory assistance than those with admission glucose less than 200mg/dL without the numbers achieving statistical significance.

**Table 4:** Associations between glycaemic control and need for ventilator support in patients with T2DM and COVID-19 § p computed by chi-square test. p<0.05 is considered statistically significant. Fisher’s exact approach for post hoc analysis of a chi-squared test. a - difference between Groups I and II, b - difference between Groups I and III, c - difference between Groups I and IV, d - difference between Groups II and III, e - difference between Groups II and IV, f - difference between Groups IV and III T2DM: type 2 diabetes mellitus

Group	Ventilatory support		Total N (%)	P (Chi-square test)	Post hoc test
	No ventilation N (%)	Ventilation N (%)			
Known T2DM + HbA1c<7.5% + Admission glucose<200 mg/dL (I)	29 (90.6)	3 (9.4)	32 (100)	<0.001*	a-0.870
					b-0.042*
					c<0.001*
Known T2DM + HbA1c<7.5% + Admission glucose≥200 mg/dL (II)	30 (83.3)	6 (16.7)	36 (100)		d-0.016*
Known T2DM + HbA1c≥7.5% + Admission glucose<200 mg/dL (III)	26 (55.3)	21 (44.7)	47 (100)		
Known T2DM + HbA1c≥7.5% + Admission glucose ≥200 mg/dL (IV)	28 (45.2)	34 (54.8)	62 (100)		e-0.001*
					f-0.687
Total	113 (63.8)	64 (36.2)	177 (100)		

## Discussion

In this retrospective study, we examined the effect of glycaemic control on the prognosis of COVID-19 in patients with T2DM. We considered both random admission glucose <200 mg/dL and HbA1c <7.5% as a criterion of good glycaemic control over the past three months and immediately, respectively. Few other observational studies concentrated on blood glucose control for the prognosis of COVID-19 patients with diabetes however they used only blood glucose level as a surrogate of glycaemic control [7,14,18-20]. Bhandari S et al. conducted a similar study but they categorized patients into two groups based on an HbA1c cut-off of less than 8% [21]. Our results show that as a combined group, patients with poorly controlled diabetes presented to the hospital at an older age and progressed to severe COVID-19 which is in line with the report of risk factors for severe COVID-19, with the strongest associations found for age, obesity, and diabetes [14,18].

The presence of diabetes mellitus and the individual degree of hyperglycemia seems to be independently associated with COVID-19 severity and increased mortality [22-24]. The present study also confirms this finding. Patients with poor glycaemic control were found to have higher chest CTSS indicating extensive lung involvement and subsequently higher requirement for ventilatory support. The association of glycaemic control and disease severity remained significant even after adjustment for age and body weight. However HbA1c level ≥7.5% was a better predictor for a worse prognosis than admission plasma glucose in terms of length of stay in the hospital and need for supportive therapies like ICU care and need of mechanical ventilation.

Last, the primary endpoint of our study was in-hospital death. The overall mortality in our study was 10.17%. However, no significant difference was observed in mortality rates between the well-controlled and poorly controlled patients. Raoufi M et al. collected clinical characteristics of 117 patients with coexistent COVID-19 and diabetes using HbA1c as an index of glucose management and reported similar findings [25]. Li Y et al. included 132 patients with COVID-19 and diabetes and suggested patients with admission glucose >11mmol/L had an increased risk of mortality and in-hospital complications [26].

Diabetes mellitus has already been a leading cause of morbidity worldwide and is capable of affecting almost every system of the body. Consequently, a dysregulated immune system might develop; predisposing to various infections in patients with T2DM [27].

Although this study generated a significant suggestion for glucose management for COVID-19 patients with type 2 diabetes, several limitations should be addressed. First, the study was conducted during a pandemic setting when the simultaneous influx of a large number of patients jeopardized the healthcare system. Since conducting a randomized controlled in this situation could have been unethical hence the retrospective study was carried out. Our study protocol was designed for descriptive analysis and it was beyond the scope of this assessment to quantify the magnitude of the association between diabetes and glycaemic control and treatment outcomes. Second, as hospitalized patients were only included in the study these results cannot be directly extrapolated to patients with milder diseases. Third, our dataset did not include recognized comorbidities for death from COVID-19, such as hypertension or cardiovascular disease. Fourth, given the retrospective nature of the study, it was not possible for us to determine if active management of blood glucose levels to a more normal range could ameliorate COVID-19 severity or adverse outcomes. Another limitation of this study includes the lack of differentiation between the duration of the disease. Lastly, data collected from a single center and thus not very large in number exposes the statistical analysis to selection bias and the results may be less efficacious from a statistical standpoint. Therefore, large-scale prospective cohort studies will be required in ethnically and geographically diverse cohorts to better understand the association and importance of glycaemic control in the disease progression of COVID-19.

## Conclusions

The present study showed that neither HbA1c nor admission glucose values could predict mortality in patients with T2DM suffering from COVID-19 but within the same range of HbA1c, those with admission glucose less than 200mg/dL had numerical supremacy in terms of less mortality which failed to reach statistical significance. CTSS and hence lung involvement was significantly worse in those with high HbA1c and high admission glucose in comparison to those having controlled HbA1c and admission glucose values. High HbA1c was significantly associated with a longer duration of hospital stay and ICU stays, however, amongst those with high HbA1c higher admission glucose significantly increased the duration of ICU stay. Keeping in mind the high prevalence of diabetes in India, it predisposes a large proportion of the population to COVID-19 and its complications. Our results provided a simple and practical way to risk stratify COVID-19 in-patients with T2DM for hierarchical management, particularly where medical resources are in severe shortage during the pandemic provided a larger number of studies in future revalidate the findings.
